# Dynamic evaluation of the ecological civilization of Jiangxi Province: GIS and AHP approaches

**DOI:** 10.1371/journal.pone.0271768

**Published:** 2022-08-04

**Authors:** Wei Xu, Jiahui Yi, Jing Shuai, Zhen Yu, Jinhua Cheng

**Affiliations:** 1 School of Economics and Management, China University of Geosciences, Wuhan, China; 2 School of Economics and Management, East China University of Technology, Nanchang, China; Northeastern University (Shenyang China), CHINA

## Abstract

Faced with the increasingly severe ecological environment, China promotes the construction of ecological civilization vigorously. Therefore, it is of great significance to adopt scientific, effective and comprehensive methods to evaluate development status of ecological civilization. Based on the panel data from 2010 to 2014, this paper employs GIS and AHP methods to dynamically examine the level of ecological civilization construction in Jiangxi Province. The results indicate that: (1) The ecological civilization construction in Jiangxi Province is 13.23% higher than the national average, whereas there is an imbalance in the development of different cities within the province; (2) The ecological civilization construction in the whole province rises first and then falls; (3) The performances of the cities vary in different dimensions of the construction of ecological civilization that cities in Jiangxi province perform well in the dimension of ecological environment, but perform poorly in the dimension of social development. Finally, we put forward policy recommendations for improving ecological environment to realize harmonious development between human and nature.

## Introduction

**Fig 1:**
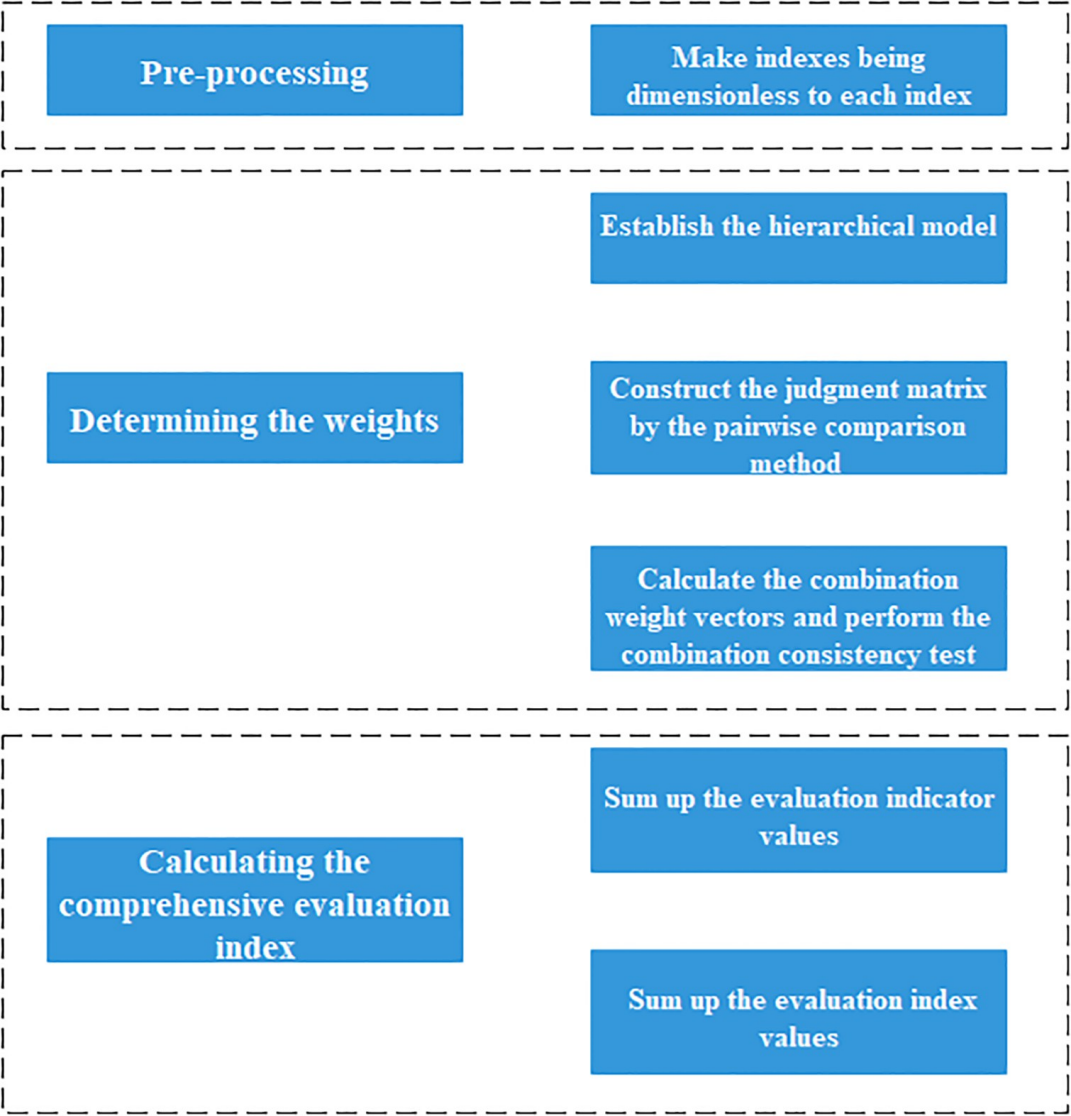
The workflow of evaluation methods.

The construction of ecological civilization has been put forward in the 17th National Congress of the Communist Party of China (CPC) and become one of target on building moderately prosperous society. Since then, it has also been listed into the "five in one" socialist construction in the overall layout of the 18th CPC Congress. Facts show that promoting the construction of ecological civilization plays a significant and far-reaching role to achieving sustainable development for the new era of China. Jiangxi Province, located in the important strategic position of mid-China, which links the north and south, the east and the west of China, is a land of abundance with a good ecological environment. Central leaders attach great importance to the construction of ecological civilization in Jiangxi Province, they instruct repeatedly to build Jiangxi in a good way to keep the mountains green and Poyang Lake clean. The domestic ecological civilization construction evaluation theory and practice mode has also gradually developed in the national ecological civilization construction environment. Scientific evaluation index system of ecological civilization could not only evaluate ecological civilization level, but also provide targeted policy recommendations and decision- reference [1, 2]. Therefore, it is of great significance to carry out the scientific, effective and comprehensive dynamic evaluation of the construction of ecological civilization in Jiangxi Province, and to promote the further development of ecological civilization in Jiangxi Province. The AHP (Analytic Hierarchy Process) method can analyze the collected data by identifying and weighting selection criteria and use quantitative and qualitative criteria for joint evaluation thereby simplifying and systematizing the complex decision-making process [3]. Meanwhile, GIS (Geographic Information System) can process and analyze data with different attribute content quickly, efficiently and flexibly [4]. This paper adopts the method of combining AHP and GIS to determine which criteria should be considered in the evaluation of ecological civilization construction and the dynamic spatial distribution of ecological civilization construction level within the same area, so as to identify the development degree of ecological civilization. The novelty of this paper can be summarized as follows: (1) Through in-depth research on relevant literature and industry reports, a standard system (resources, ecological environment, economic efficiency, social development standards and their sub-standards) for further evaluation of ecological civilization construction is established. (2) The use of the integration of GIS-AHP to coordinate the important roles and priorities of different dimensions in the construction of ecological civilization has reference significance for formulating differentiated and regional coordinated policies. The workflow of the evaluation methods is given in Fig 1.

## Literature review

After industrial civilization, ecological civilization is a new stage of human civilization in which the aim is to achieve "harmonious growth between man and environment" [5]. Griggs et al. (2013) [6] propose six sustainable development goals: prosperous living, safe food, secure water, clean energy, a healthy ecology, and stable social governance. Kerk et al. (2008) [7] evaluates a country’s long-term viability based on five factors: personal growth, environmental cleanliness, social development, resource exploitation, and global viability. Meanwhile, many researchers are concentrating their efforts on urban areas [8–14]. Fritz et al. (2016) [15] believe that economic expansion fosters social and human development while also causing the ecological environment to become unsustainable. Environmental poverty, as defined by Liu (2012) [16], is a lack of a healthy environment for society’s survival and growth as a result of induced environmental deterioration, with the effect being significantly bigger in low- and middle-income nations than in low- and high-income countries [17]. Though the link between economy and the environment in China has improved significantly, the rate of improvement is slower than the rate of economic expansion [18, 19]. Furthermore, a variety of factors, including politics, population, science and technology, industrial structure, and others, will have an impact on China’s environmental sustainability, and any individual power will have a direct or indirect impact on it [20–24]. Lou et al. (2013) [25] evaluates economic performance and environmental loads with the energy sustainability index (ESI). Ecological innovation is not only the key to realize sustainable economic growth, but also the fundamental way to ease the economic and environmental tensions [26, 27], As for the objective of ecological civilization construction, Yu et al. (2016) [28] believes the establishment of Poyang Lake ecological economic zone could promote ecological innovation effectively. While evaluating the level of ecological innovation in the region, Chen et al. (2017) [27] considers that the technology is driven by the market and the environmental regulation has a positive impact on it. China faces many challenges in the process of urbanization and industrialization, which includes land, water, energy and environment, and they are all related to carbon dioxide emissions [29–32], The construction of ecological civilization can not only increase the capital efficiency of low-carbon economy development, but also enhance the competitiveness of low-carbon economy development [33], China should incorporate it into the whole process of urbanization and industrialization [31].

The energy sustainability index, developed by Lou et al. (2013) [25], assesses economic performance and environmental burdens (ESI). Ecological innovation is not only essential for achieving long-term economic growth, but also for reducing environmental pressures (Arkar, 2013; 27), In terms of the goal of ecological civilization development, Yu et al. (2016) [28] believes that Poyang Lake ecological economic zone will successfully encourage ecological innovation. Chen et al. (2017) [27] believes that the technology driven by market and environmental regulation has a beneficial influence on innovation in turn. In the process of urbanization and industrialization, China faces many challenges in the process of urbanization and industrialization, including land, water, energy, and the environment, all of which are linked to carbon dioxide emissions [29–33]. The development of ecological civilization may improve not only the capital efficiency of low-carbon economy growth, but also its competitiveness [34]. China should include it throughout the whole process of urbanization and industrialization [31].

Currently, the evaluation system of ecological civilization construction is mainly developed on two aspects-theoretical research and practical exploration [35]. Theoretical research mainly focused on the national, provincial and city level, whereas the practical exploration is to construct evaluation system according to the specific research objects, and the construction of a comprehensive indicator must consider the connection between each system and its dynamic development [36]. Yan et al. (2013) [37] calculates the ecological civilization index (ECI) and the green ecological civilization index (GECI) of the provinces based on the improved system in the provinces of China (ECCI2013). Mi et al. (2016) [38] constructs the system in China from three perspectives of nature, economy and society. Zhang et al. (2014) [39] constructs the provincial system of evaluation index system of ecological system pressure, ecological system health status and ecological environment management in the pressure-state-response (PSR) model; According to megacity’s feature, Zhang et al. (2015) [40] constructs it from four aspects, such as ecological environmental health, resource and environmental consumption intensity, pollution control efficiency of surface source and habitability of residents, which also takes Wuhan city as application example; Cheng et al. (2013) [41] optimizes his system specifically with mining area. Qin et al. (2013) [42] put forward six—dimensional roadmap of ecological civilization city construction, and they construct its evaluation index system, covering system guarantee, ecological habitat, environmental support, economic operation and consciousness culture. Liu et al. (2014) [43] select the ecosystem service function, ecological footprint and per capita GDP to reflect the resource endowment, and comprehensively evaluate the ecological civilization construction in all provinces and cities across the country. Li et al. (2016) [44], Hu et al. (2015) [45] focus on efficiency evaluation of ecological civilization construction. In which, main research methods include principal component analysis, analytic hierarchy process, entropy weight method, TOPSIS method, network analytic hierarchy process, set analysis method, ecological footprint method [46, 47] data envelopment analysis etc., and using tools such as GIS to improve the visualization of research results [48]. Besides it, some scholars research coupling relationship between ecological civilization and other fields. Bi et al. (2017) [49] think the coupling coordination and development between china ecological civilization construction and urbanization lies in run-in period, whereas the ecological civilization construction develops less than urbanization level. Li et al. (2015) [50] believe that China’s ecological civilization construction level continues to improve, and the coordination and development capacity between the environment, economy and society is constantly strengthened. Yao and Yang (2017) [51] identify the key factors of ecological civilization construction and find that the ecological civilization system occupies a higher and higher proportion in the key factors. Ma et al. (2016) [52] argues that the main factors influencing the level of urban agglomeration in the middle reach of Yangtze River ecological civilization are per capita GDP, per capita residents social retail sales of consumer goods and services the added value of a share of GDP, per capita water resources, urban residents’ per capita disposable income.

From the above review that a series of researches have been conducted in China in the ecological civilization construction evaluation in recent years, and achieved preliminary research results. At present, the scholars use the AHP evaluation method to construct the evaluation index system of ecological civilization construction from each way. They determine the weight through expert scoring method and calculate the level of ecological civilization construction in each region, then evaluate the development of ecological civilization construction levels. However, there is also some research gaps in this area: (1) There is a lack of dynamic evaluation: the existing research belongs to the static evaluation, the dynamic research on ecological civilization construction has not yet been carried out. For the provincial ecological civilization construction evaluation, scholars usually assess the prefecture city of a year, and rarely cover the entire province of the prefecture cities with continuous evaluation; (2) There is a lack of spatial comparison: the existing research methods are relatively simple and lack spatial comparative analysis within the same region. At present, the research literature on the evaluation of the construction level of ecological civilization is mostly based on the AHP method to evaluate it at a certain point in a certain region. It has not yet been carried out on the combination of AHP and GIS research on Dynamic Evaluation of Horizontal Level. The GIS technique is used to visualize the level of ecological civilization construction, so as to achieve cross-spatial units and different dimensions of ecological civilization construction, thereby providing more information for the ecological civilization construction policies. The integration of GIS and AHP methods combines qualitative and quantitative assessment with spatial data to dynamically analyze the multi-dimensional spatiotemporal changes of ecological civilization construction. Therefore, this paper first constructs the evaluation system of ecological civilization construction level of Jiangxi province with four indexes, and uses AHP to determine the weight of evaluation index. By calculating the comprehensive evaluation index, the spatial distribution of the level of ecological civilization construction in Jiangxi Province is analyzed and revealed by GIS tools. The dynamic evaluation of ecological civilization construction level based on AHP and GIS method has been carried out to fill the shortcomings of this research field.

Currently, the assessment method of ecological civilization is based primarily on two aspects: theoretical research and actual investigation (Huang et al., 2015). Theoretical research has primarily focused on national, provincial, and city levels, whereas practical exploration focuses on constructing evaluation systems based on specific research objects, and the development of a comprehensive indicator must take into account the relationship between each system and its dynamic development (Singh et al., 2012). Yan et al. (2013) calculates the ecological civilization index (ECI) and the green ecological civilization index (GECI) of the provinces based on the improved system in the provinces of China (ECCI2013). Bi et al. (2016) examines China’s system from three angles: nature, economics, and society. In the pressure-state-response (PSR) model, Zhang et al. (2014) designs the provincial system of assessment index system of ecological system pressure, ecological system health status, and ecological environment management. Zhang et al. (2015) constructs a megacity from four aspects, including ecological environmental health, resource and environmental consumption intensity, pollution control efficiency of surface sources, and resident habitability, with Wuhan city as an example; Cheng et al. (2013) optimizes his system specifically with mining area. Qin et al. (2013) proposes a six-dimensional roadmap for the creation of an ecological civilization city, as well as an assessment index system that includes system assurance, ecological habitat, environmental support, economic operation, and awareness culture. To represent the resource endowment, Liu et al. (2014) selects the ecosystem service function, ecological footprint, and per capita GDP, and analyze the ecological civilization construction in all provinces and cities throughout the nation. Li et al. (2016), Hu et al. (2015) focus on efficiency evaluation of ecological civilization construction. Principal component analysis, analytic hierarchy process, entropy weight method, TOPSIS method, network analytic hierarchy process, set analysis method, ecological footprint method (Borucke et al., 2013; Guo et al., 2016), data envelopment analysis, and other research methods are used, as well as using tools like GIS to improve the visualization of research results (Lu et al., 2016). Aside from that, several research investigates the connections between ecological civilization and other subjects. According to Bi et al. (2017), the coordination and growth of China’s ecological civilization construction and urbanization is still in the early stages, with ecological civilization construction progressing at a slower pace than urbanization. According to Li et al. (2015), China’s ecological civilization construction level is improving, and the coordination between the environment, economy, and society is consistently enhanced. The essential variables of ecological civilization construction are identified by Yao and Yang (2017), who concludes that the ecological civilization system occupies an increasing share of the important components. According to Ma et al. (2016), per capita GDP, per capita residents social retail sales of consumer goods and services the added value of a share of GDP, per capita water resources, urban residents’ per capita disposable income are the main factors influencing the level of urban agglomeration in the middle reach of the Yangtze River ecological civilization.

According to the above study, a series of investigations on the ecological civilization construction assessment have been done in China in recent years, with preliminary research outcomes. Scholars are now using the AHP assessment approach to build an evaluation index system for ecological civilization creation from all angles. They compute the degree of ecological civilization construction in each area and then assess the progress of ecological civilization construction levels using an expert scoring technique. There are, however, certain research gaps in this area: (1) The current study is a static assessment; no dynamic research on the ecological civilization construction has yet been conducted. Scholars generally evaluate the prefecture city of a year for the provincial ecological civilization construction assessment, and it is uncommon to cover the whole province of prefecture cities with continuous evaluation; (2) the present research methodologies are rather basic. At the moment, the majority of the study literature on the assessment of ecological civilization’s construction level is based on the AHP approach to assess it at a specific place in a specific area. There hasn’t been any study on Dynamic Horizontal Level Evaluation using a mix of AHP and GIS yet. As a result, this study first creates a four-index assessment system for Jiangxi province’s ecological civilization construction level, then utilizes AHP to calculate the weight of each evaluation indicator. The geographical distribution of the degree of ecological civilization construction in Jiangxi Province is investigated and exposed using GIS technologies by computing the complete assessment index. To address the study field’s inadequacies, a dynamic assessment of ecological civilization construction level was conducted using the AHP and GIS methods.

## Building an ecological civilization evaluation index system for Jiangxi Province

It is required to scientifically describe the main factors in ecological civilization building and propose policy-making recommendations for Jiangxi’s assessment index system of ecological civilization construction level. As a result, this paper constructs it in four aspects, namely, resource conditions, ecological environment, economic efficiency, and social development, based on the research results of the existing system and following the principles of integrity, orientation, operability, quantitative, and policy-making (see Table 1).

**Table 1:** Evaluation index system of the ecological civilization of Jiangxi Province.

Target Layer	Criteria Layer	Indicator Layer	Computing Method	Attribute	Evaluation Significance
The general target of ecological civilization construction in Jiangxi province	Resources conditions	Per capita arable land (Mu/person)	Statistical indicators	+	To evaluate resources situation of arable land, water, forest and employees
		per capita water resources (m^3^/person)	Statistical indicators	+	
		Forest cover rate (%)	Statistical indicators	+	
		% of employees in the total population (%)	CP/total population	+	
	Ecological environment	Built green park green area (%)	Statistical indicators	+	To evaluate urban greening
		Per capita park green area (m^2^/person)	Park green gross area/population	+	
		% of environmental protection expenditure to fiscal budget (%)	environmental protection expenditure/fiscal budget	+	To evaluate environmental protection input
		Sulfur dioxide emissions (kg/ten-thousand-yuan output value)	Sulfur dioxide emissions/GDP	-	To evaluate three-waste emission intensity and its treatment efficiency
		Harmless treatment rate of household garbage (%)	Statistical indicators	+	
		Comprehensive utilization rate of industrial solid waste (%)	Statistical indicators	+	
		Centralized treatment rate of urban sewage (%)	Statistical indicators	+	
	Economic efficiency	Average output value of designed size enterprises (billion/unit)	Total output value of designed size enterprises/Total designed size enterprises	+	To evaluate economy’s scale benefit, scientific and technological efficiency, per capita efficiency and energy efficiency
		Per capita GDP (yuan/person)	Statistical indicators	+	
		% of output value of science and technology industry to GDP (%)	output value of science and technology industry/GDP	+	
		Energy consumption per unit (SCE/ten thousand yuan)	Statistical indicators	-	
	Social development	Urban per capita disposable income (yuan)	Statistical indicators	+	To evaluate urban and rural income level and purchasing level
		Rural per capita net income (yuan)	Statistical indicators	+	
		Residents ‘per capita retail sales of consumer goods (yuan/person)	Total retail sales of consumer goods/ population	+	
		Urban per capita housing area (m^2^/person)	Statistical indicators	+	To evaluate residents’ housing, education and medical treatment
		education fiscal expenditure to GDP (%)	education fiscal expenditure/GDP	+	
		number of doctors per thousand (person / thousand person)	Statistical indicators	+	

Data: constructed by practical situation of ecological civilization development in Jiangxi Province.

A diversity of resources offers a firm basis for the creation of ecological civilization in terms of resource circumstances. Land resources, water resources, forest resources, and human resources are the four kinds of resources in general. Land, water, and forest resources are quickly and heavily depleted as a result of industrialization. When the availability of resources is insufficient to fulfill the demands of industrial growth, social and economic progress will be stifled. As a result, this article assesses resource conditions using four indicators: per capita arable land, per capita water resources, forest coverage rate, and employee-to-population ratio.

In terms of the ecological environment, ecological civilization creation starts with ecological environment construction (Huang et al., 2015). Controlling pollutant and waste emissions, as well as preserving a healthy ecological environment, may help to foster economic and social growth that is both sustainable and healthful. In general, three groups make up the urban ecological environment: urban ecosystems, environmental emissions, and "three waste management." As a result, the ecological environment is assessed using seven indicators: built green coverage rate, per capita park green area, proportion of environmental protection expenditure to fiscal budget, scale of sulfur dioxide emissions, Harmless treatment rate of household garbage, comprehensive utilization rate of industrial solid waste, and centralized treatment rate of urban sewage.

In terms of economic efficiency, this is represented not only in the rise in production per unit of material consumption, but also in the increase in per capita output, all of which indicates that less investment is required in return for more output. In general, there are four types of economic advantages: scale benefits, per capita benefits, scientific and technology benefits, and energy efficiency benefits. As a result, four metrics are used to assess the economic benefits: average output value of Designed Size Enterprises, per capita GDP, percentage rate of output value of science and technology sector to GDP, and unit GDP energy use.

Stable social development is favorable to the establishment of ecological civilization in terms of social development. Household income, household consumption, residents’ housing, medical level, and educational level are the five factors that make up social development in general. As a result, this article uses six indicators to assess social development: urban per capita disposable income, rural per capita net income, residents’ per capita retail sales of social consumer goods, urban per capita housing area, education fiscal expenditure as a percentage of GDP, and the number of doctors per thousand.

## Data sources and evaluation methods

### Data sources and pre-processing

The study evaluates ecological civilization construction level of Jiangxi Province and its 11 prefecture-level cities in 2010–2014. The data are mainly derived from the statistical yearbook of Jiangxi Provincial Bureau of Statistics (http://tjj.jiangxi.gov.cn/col/col38595/index.html). Meanwhile, with the national ecological civilization construction level in same year as the reference value, this article makes indexes being dimensionless to each index of observation. The specific formula are as follows:

$$\begin{array}{ll} \text{Positive}\;\text{dimension}: & y_{ij}=(x_{ij}/B_{ij})\times 100 \end{array}$$ (1)

$$\begin{array}{ll} \text{Negative}\;\text{dimension}: & y_{ij}=(B_{ij}/x_{ij})\times 100 \end{array}$$ (2)

*where*, *x*_*ij*_ works as the *i-th* index and observed value of the *j-th* evaluation object, *B*_*ij*_ *w*orks as the *i-th* index and national average value of the *j-th* evaluation object, and *y*_*ij*_ works as the *i-th* index and dimensionless value of the *j-th* evaluation object. Taking the year of 2014 as an example, after dimensionless method, the evaluation value of Jiangxi Province’s ecological civilization construction level is as shown in Table 2. And we assume that China’s average level is 100 points.

**Table 2:** Indicator values of ecological civilization of Jiangxi Province after dimensionless (2014).

Indicator Layer	Jiangxi	Nanchang	Yingtan	Xinyu	Yichun	Ganzhou	Fuzhou	Jiujiang	Ji’an	ShangRao	PingXiang	JinDeZhen
Per capita arable land	68.90	54.04	81.06	4.46	45.26	39.18	87.14	51.34	91.86	57.41	81.73	52.69
per capita water resources	180.15	69.08	202.13	147.28	198.36	160.97	281.12	158.74	238.72	209.20	118.38	166.61
Forest cover rate	292.13	101.67	265.65	261.53	298.61	352.96	298.80	254.26	313.01	285.51	305.65	301.25
% of employees in the total population	101.48	111.54	119.26	98.99	106.49	110.80	98.80	114.19	101.78	113.28	108.76	111.47
Built green park green area	110.82	104.68	102.84	126.37	108.21	99.33	116.67	127.04	113.81	116.12	100.77	128.48
Per capita park green area	107.86	91.91	101.45	138.02	118.70	79.16	125.04	134.73	129.54	108.93	80.76	113.05
% of environmental protection expenditure to fiscal budget	69.80	33.48	226.35	70.63	84.94	59.08	115.13	80.55	88.14	77.45	122.17	50.23
Sulfur dioxide emissions	94.30	307.39	103.07	51.48	72.38	111.44	163.34	69.36	110.45	135.85	30.50	78.09
Harmless treatment rate of household garbage	101.41	108.93	108.93	108.93	108.93	108.93	108.93	108.93	108.93	108.93	108.93	108.93
Comprehensive utilization rate of industrial solid waste	91.00	154.43	139.11	144.17	144.69	132.17	143.69	97.25	157.18	30.55	156.34	158.97
Centralized treatment rate of urban sewage	91.52	101.97	104.46	107.32	103.30	45.55	102.21	108.28	100.84	100.13	94.11	79.90
Average output value of large-scale enterprises	120.44	159.02	328.11	176.09	114.26	94.74	61.55	140.75	98.06	119.33	86.09	122.91
Per capita GDP	74.22	150.17	138.01	166.39	59.48	46.49	55.93	79.44	54.59	49.73	98.18	97.18
% of output value of science and technology industry to GDP	218.85	148.44	177.42	223.77	235.20	204.36	120.06	292.42	326.12	203.95	382.29	176.66
Energy consumption per unit	148.26	214.27	237.50	71.29	128.21	188.35	191.24	123.64	225.05	183.40	73.39	153.38
Urban per capita disposable income	82.74	99.01	83.70	94.03	79.03	78.06	78.63	85.35	84.40	83.92	88.56	90.62
Rural per capita net income	102.21	125.42	114.67	129.63	106.34	70.18	105.17	102.43	93.57	91.96	129.01	116.66
Residents ‘per capita retail sales of consumer goods	58.62	141.58	68.15	85.46	43.22	38.42	49.54	53.61	36.24	42.55	73.21	76.41
Urban per capita housing area	124.62	97.60	129.18	120.06	152.31	138.78	91.67	127.39	129.97	125.26	108.91	104.16
education fiscal expenditure to GDP	124.99	61.25	58.92	58.80	125.58	162.89	136.53	110.98	149.34	155.03	74.96	80.00
number of doctors per thousand	64.62	111.32	99.53	99.53	65.09	60.38	58.96	88.21	70.28	75.00	104.25	84.43

### Evaluation methodologies

We use AHP method to calculate the weights of each indicator, and then construct the comprehensive evaluation index to evaluate the level of ecological civilization construction in Jiangxi Province from 2010 to 2014.

① Determining the weights. Firstly, we establish the hierarchical model according to the above indicators. Secondly, we construct the judgment matrix by the pairwise comparison method and the 1–9 comparison scale based on the expert scoring. Then, we calculate the weight vectors and perform the consistency test. And finally, we calculate the combination weight vectors and perform the combination consistency test. According to the survey results of 13 experts, we got the weights of each ecological civilization indicator in Jiangxi Province (as shown in Table 3).

**Table 3:** Indicator weights of the ecological civilization of Jiangxi Province.

Criteria layer	Weight	Indicator layer	Weight
Resource condition	0.1431	Per capita arable land	0.0418
		Per capita water resources	0.0276
		Forest cover rate	0.0522
		% of employees in the total population	0.0215
Ecological environment	0.4706	Built green coverage rate	0.0429
		Per capita park green area	0.0341
		% of environmental protection expenditure to fiscal budget	0.1333
		Sulfur dioxide emissions	0.0541
		Harmless treatment rate of household garbage	0.0657
		Comprehensive utilization rate of industrial solid waste	0.0667
		Centralized treatment rate of urban sewage	0.0738
Economic efficiency	0.1377	Average output value of industrial enterprises above designated size	0.0150
		Per capita GDP	0.0258
		% of output value of science and technology industry to GDP	0.0606
		Energy consumption per unit of GDP	0.0363
Social development	0.2486	Per capita disposable income of urban residents	0.0408
		Per capita net income of farmers	0.0467
		Residents’ per capita retail sales of social consumer goods	0.0293
		Per capita housing area of urban residents	0.0221
		% of education financial expenditure to GDP	0.0587
		Number of doctors per thousand people	0.0510

Source: calculated by the AHP method according to the expert scoring data.

② Calculating the comprehensive evaluation index. According to the indicator weights calculated through AHP method, we construct the comprehensive ecological civilization evaluation index. Firstly, we sum up the evaluation indicator values after dimensionless processing at the indicator layer according to the corresponding objective weights, and then we acquire the corresponding evaluation index values at the criterion layer. Secondly, we sum up the evaluation index values according to the corresponding objective weights and get the evaluation result of the ecological civilization construction level in Jiangxi province and its prefecture-level cities from 2010 to 2014. The specific formulas are as follows:

$$v_{j}=\sum_{i=1}^{n}y_{i}w_{i}$$ (3)

$$V=\sum_{j=1}^{n}v_{j}w_{j}$$ (4)

In which, *y*_*i*_ refers to the dimensionless value of the i-th indicator, *v*_*j*_ refers to the evaluation value of the j-th criterion, *V* refers to the evaluation index of the ecological civilization construction level, and *w*_*i*_, *w*_*j*_ refer to the weights of i-th indicator and the j-th criterion respectively.

## Results

### Comprehensive evaluation results

According to the above evaluation methodologies, we obtain the evaluation results of ecological civilization construction level in Jiangxi province and its prefecture-level cities from 2010 to 2014 (as shown in Table 4).

**Table 4:** Evaluation results of ecological civilization of cities in Jiangxi Province.

Ecological Civilization	Nanchang	Yingtan	Xinyu	Jingdezhen	Ganzhou	Fuzhou	Jiujiang	Ji’an	Shangao	Pingxiang	Yichun	Total
2010	118.29	126.80	122.85	119.05	119.16	111.91	111.98	118.26	110.10	120.95	108.75	113.95
2011	118.85	125.44	129.40	126.94	112.93	109.09	106.15	123.82	112.92	119.48	106.57	113.90
2012	114.47	129.72	154.54	125.26	116.04	120.27	110.86	128.56	116.22	126.28	112.87	117.23
2013	113.60	144.72	134.27	126.07	114.11	125.31	114.47	133.98	116.27	126.22	116.62	114.58
2014	114.08	143.17	113.71	112.18	112.22	126.06	118.46	135.97	115.21	128.08	119.27	113.23
Resources Qualification	Nanchang	Yingtan	Xinyu	Jingdezhen	Ganzhou	Fuzhou	Jiujiang	Ji’an	Shangao	Pingxiang	Yichun	Total
2010	12.28	27.00	20.44	26.60	28.31	30.23	23.37	28.80	26.25	24.50	23.10	26.34
2011	11.39	24.09	18.20	24.52	26.35	24.83	21.26	26.54	24.36	23.37	21.18	23.94
2012	12.16	27.56	20.53	25.86	28.97	30.35	23.08	28.85	25.59	24.71	28.38	26.37
2013	11.65	25.05	18.71	24.46	27.13	27.12	21.64	27.65	24.57	23.85	24.12	24.51
2014	11.87	25.40	20.03	24.92	26.89	29.12	22.25	28.96	25.51	24.98	25.24	25.28
Eco- environment	Nanchang	Yingtan	Xinyu	Jingdezhen	Ganzhou	Fuzhou	Jiujiang	Ji’an	Shangao	Pingxiang	Yichun	Total
2010	60.06	52.29	54.48	48.82	54.42	45.75	52.87	48.71	47.39	49.01	50.29	46.28
2011	60.66	50.64	57.72	56.19	45.20	47.47	45.81	52.15	47.94	47.69	46.94	45.44
2012	55.92	50.57	88.32	56.23	46.34	52.36	45.66	52.92	47.10	50.82	45.42	46.37
2013	54.37	69.46	70.72	52.18	43.03	59.73	46.21	54.35	45.48	49.30	50.53	44.91
2014	53.70	67.76	47.02	43.94	40.20	57.74	46.17	52.11	42.95	49.54	48.36	42.32
Economic Efficiency	Nanchang	Yingtan	Xinyu	Jingdezhen	Ganzhou	Fuzhou	Jiujiang	Ji’an	Shangao	Pingxiang	Yichun	Total
2010	21.67	27.57	25.95	22.23	16.56	14.89	15.54	19.32	14.86	25.06	14.31	19.30
2011	22.47	28.71	30.85	24.32	20.82	14.82	17.58	23.03	18.16	24.83	16.75	21.42
2012	21.87	29.57	22.67	21.39	18.78	15.54	20.38	24.10	20.78	27.09	17.54	21.18
2013	22.98	28.00	21.52	27.04	21.59	16.20	23.90	28.45	21.92	29.17	19.50	21.80
2014	23.03	27.86	23.08	20.62	21.84	16.58	26.37	30.81	22.09	29.66	22.16	22.37
Social Development	Nanchang	Yingtan	Xinyu	Jingdezhen	Ganzhou	Fuzhou	Jiujiang	Ji’an	Shangao	Pingxiang	Yichun	Total
2010	24.29	19.95	21.97	21.41	19.87	21.04	20.20	21.44	21.61	22.38	21.05	22.04
2011	24.32	22.00	22.63	21.91	20.56	21.96	21.50	22.10	22.46	23.59	21.69	23.10
2012	24.51	22.02	23.04	21.78	21.95	22.03	21.74	22.69	22.75	23.66	21.52	23.30
2013	24.60	22.21	23.33	22.40	22.35	22.26	22.73	23.53	24.31	23.90	22.45	23.37
2014	25.47	22.16	23.57	22.69	23.30	22.62	23.67	24.10	24.66	23.91	23.51	23.25

Source: calculated by the AHP method according to the statistical yearbook database from 2011 to 2015.

**Fig 2:**
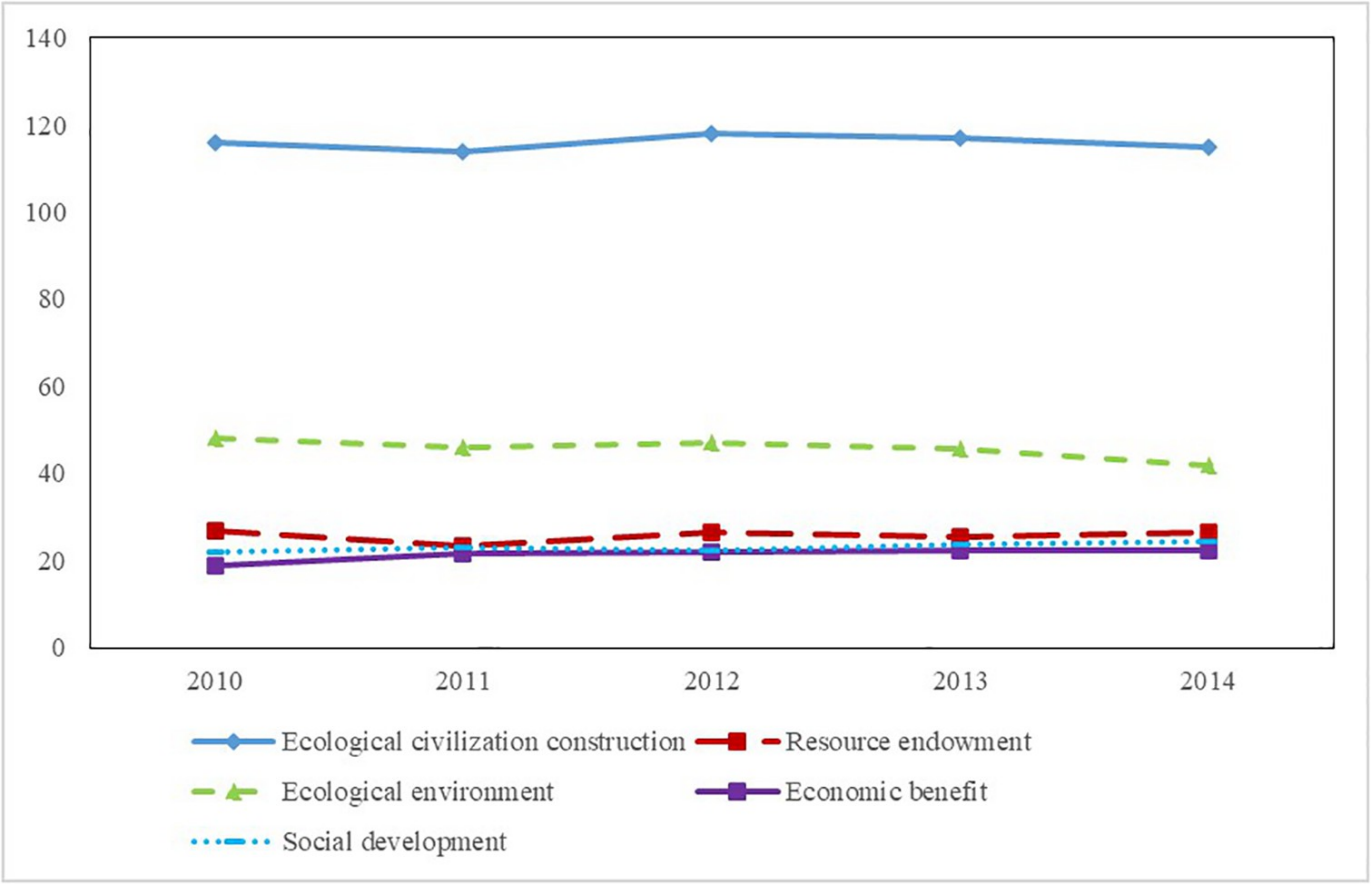
Evaluation result of the ecological civilization of Jiangxi Province. Source: Republished from Jiangxi Provincial Bureau of Statistics under a CC BY license, with permission from China Statistics Press, original copyright 2010 to 2014.

As can be seen from Table 4, the ecological civilization construction level of Jiangxi province and its 11 prefecture-level cities are higher than the average level of China from 2010 to 2014. Fig 2 shows that the ecological civilization construction level of Jiangxi province began to rise and then declined from 2010 to 2014. The highest level was in 2012.

Among the 11 prefecture-level cities, the number of cities with the ecological civilization construction level above the province’s average level gradually increases. In 2014, the number is 9, which accounted for 81.82% of the province. According to the evaluation results, the order from the high to low are as followed: Yingtan, Ji’an, Pingxiang, Fuzhou, Yichun, Jiujiang, Shangrao, Nanchang and Xinyu; only Jingdezhen and Ganzhou are lower than the province’s average level. The ecological civilization construction level of Yingtan, Xinyu, Ji’an and Pingxiang are continuously higher than the province’s average level. The ecological civilization construction level of different cities undergoes different changes during the study period (as shown in Table 5). The ecological civilization construction level of Yingtan, Fuzhou, Ji’an, Pingxiang and Yichun continues to rise; the level of Jiujiang and Shangrao rises with fluctuations; the level of Xinyu and Jingdezhen declines with fluctuations; however, the level of Nanchang and Ganzhou continues to decline.

**Table 5:** Comparison of ecological civilization between cities of Jiangxi Province and average of the whole province and China.

Year	No. higher than the national average	%	No. higher than the provincial average	%	No. lower than the provincial average	%
2010	11	100%	7	63.64%	4	36.36%
2011	11	100%	6	54.55%	5	45.45%
2012	11	100%	6	54.55%	5	45.45%
2013	11	100%	8	72.73%	3	27.27%
2014	11	100%	9	81.82%	2	18.18%

Source: compared and calculated by the ecological civilization construction level between Jiangxi and the nation.

**Fig 3:**
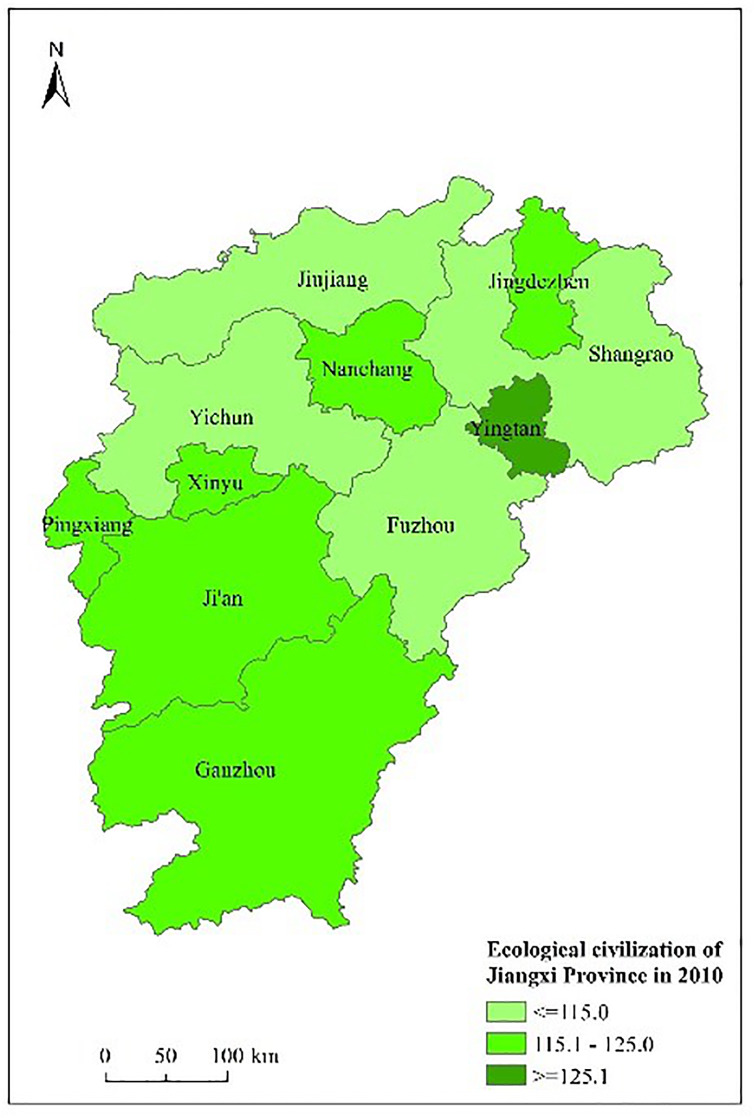
Space distribution of ecological civilization of Jiangxi Province (base map and data from OpenStreetMap and OpenStreetMap foundation. **Contains information from OpenStreetMap and OpenStreetMap foundation, which is made available under the Open Database license. Data source comes from** Table 4). Source: Republished from Jiangxi Provincial Bureau of Statistics under a CC BY license, with permission from China Statistics Press, original copyright 2010 to 2014.

**Fig 4:**
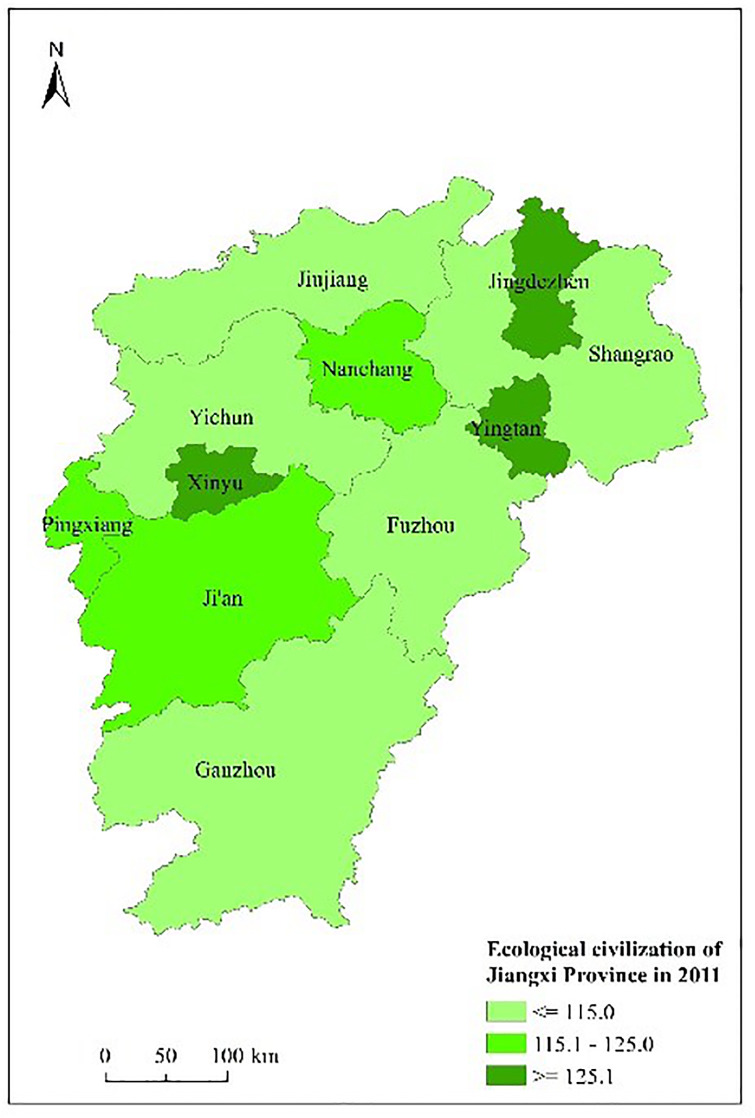
Space distribution of ecological civilization of Jiangxi Province (base map and data from OpenStreetMap and OpenStreetMap foundation. **Contains information from OpenStreetMap and OpenStreetMap foundation, which is made available under the Open Database license. Data source comes from** Table 4). Source: Republished from Jiangxi Provincial Bureau of Statistics under a CC BY license, with permission from China Statistics Press, original copyright 2010 to 2014.

**Fig 5:**
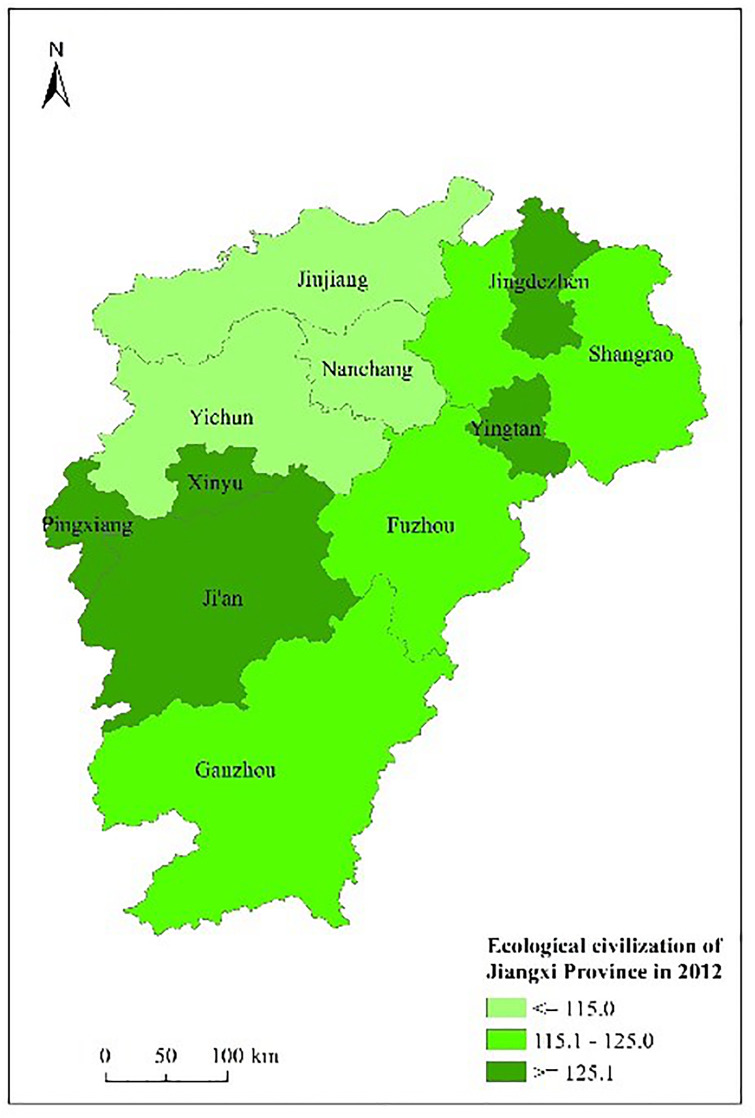
Space distribution of ecological civilization of Jiangxi Province (base map and data from OpenStreetMap and OpenStreetMap foundation. **Contains information from OpenStreetMap and OpenStreetMap foundation, which is made available under the Open Database license. Data source comes from** Table 4). Source: Republished from Jiangxi Provincial Bureau of Statistics under a CC BY license, with permission from China Statistics Press, original copyright 2010 to 2014.

**Fig 6:**
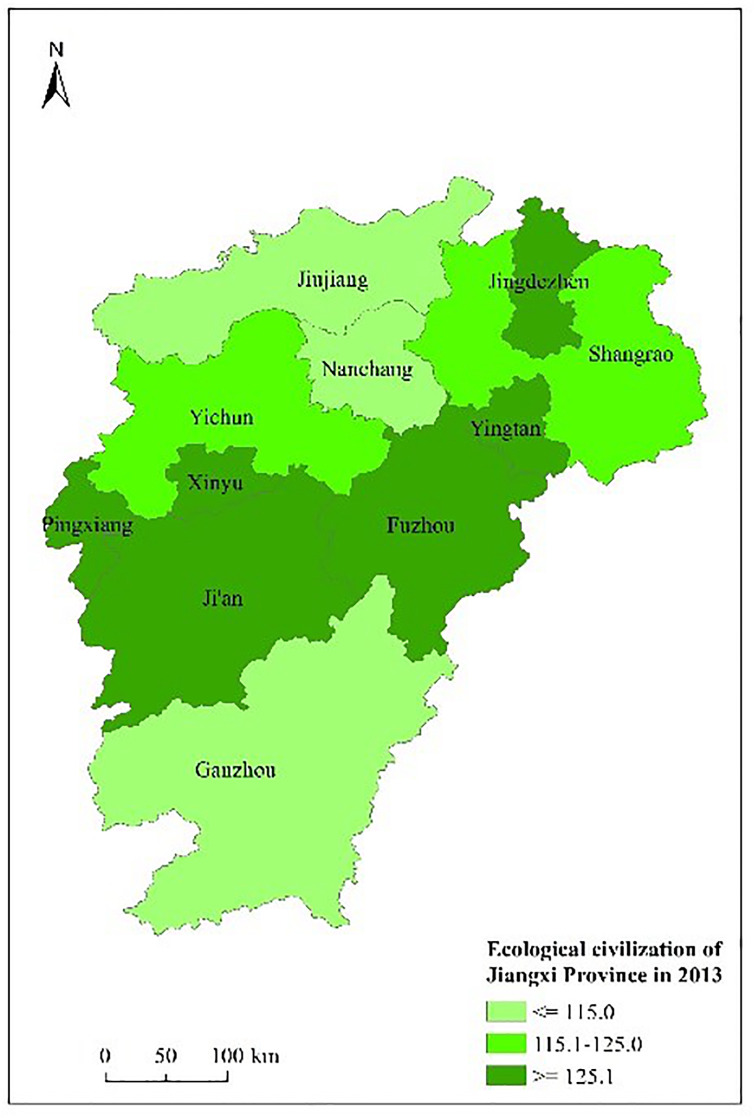
Space distribution of ecological civilization of Jiangxi Province (base map and data from OpenStreetMap and OpenStreetMap foundation. **Contains information from OpenStreetMap and OpenStreetMap foundation, which is made available under the Open Database license. Data source comes from** Table 4). Source: Republished from Jiangxi Provincial Bureau of Statistics under a CC BY license, with permission from China Statistics Press, original copyright 2010 to 2014.

**Fig 7:**
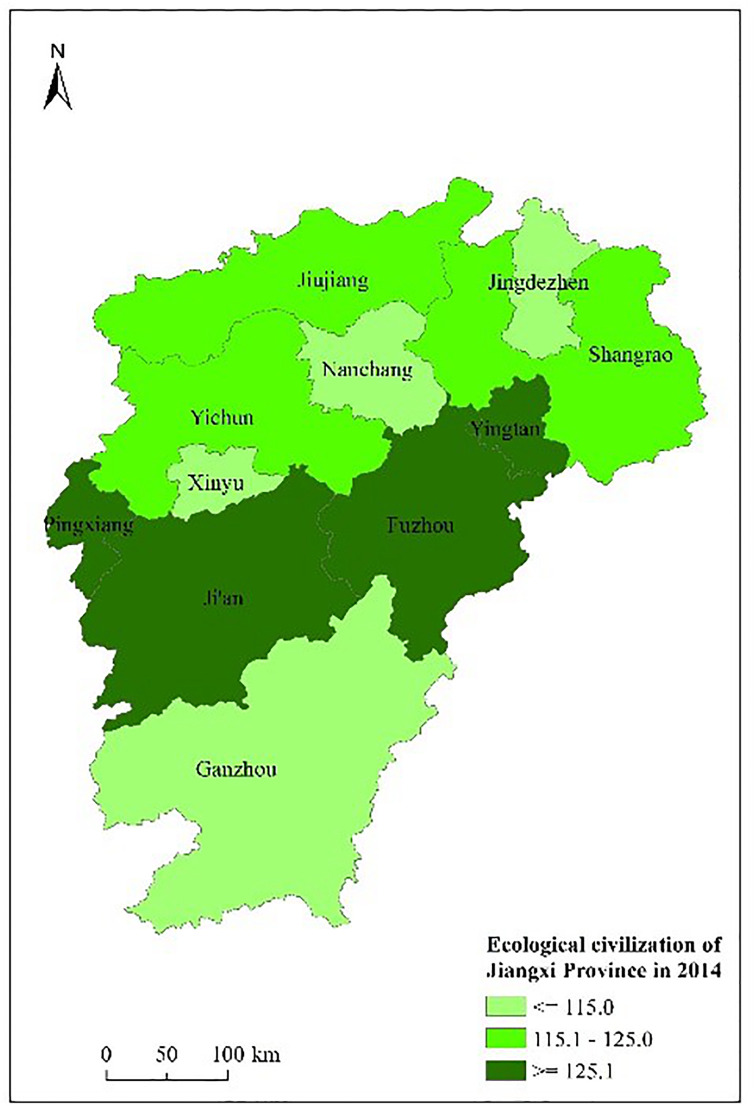
Space distribution of ecological civilization of Jiangxi Province (base map and data from OpenStreetMap and OpenStreetMap foundation. **Contains information from OpenStreetMap and OpenStreetMap foundation, which is made available under the Open Database license. Data source comes from** Table 4). Source: Republished from Jiangxi Provincial Bureau of Statistics under a CC BY license, with permission from China Statistics Press, original copyright 2010 to 2014.

Figs 3 to 7 shows the spatial distribution and its evolution of the ecological civilization construction level in Jiangxi province from 2010 to 2014. It can be seen that the number of cities with high ecological civilization construction level in Jiangxi province (i.e., the evaluation result≥125) gradually increases, and the phenomenon of spatial aggregation occurs in middle part.

According to the previous analysis, it can be seen that although more and more cities in Jiangxi province have improved their ecological civilization construction level, the overall level of Jiangxi province has declined in recent years, which indicates that although decrease of the cities with low ecological civilization construction level has a great impact on the overall level of Jiangxi province, leading to a decline in the ecological civilization construction level on the whole.

### Evaluation results at the criteria layer

Evaluation results from criterion layer show that, the ecological environment consisted in the largest proportion of ecological civilization construction level with a similar trend with the overall level, which indicates that it plays a vital role in ecological civilization in Jiangxi province (as shown in Table 6).

**Table 6:** Dynamic changes of ecological civilization of cities in Jiangxi Province.

Trend	No. of cities	%	City name
Persisting rise	5	45.46%	Yingtan, Fuzhou, Ji’an, Pingxiang, Yichun
Fluctuating rise	2	18.18%	Jiujiang, Shangrao
Fluctuating decline	2	18.18%	Xinyu, Jingdezhen
Persisting decline	2	18.18%	Nanchang, Ganzhou

Source: calculated on dynamic changes of ecological civilization in Jiangxi Province.

The resource conditions of Yingtan, Ganzhou, Fuzhou and Ji’an are with a higher endowment than the province’s average level, while the conditions of Nanchang, Xinyu, Jiujiang and Pingxiang are lower than the province’s average level. In the aspect of ecological environment, most of cities had higher level than the province’s average, whereas the ecological condition of Ganzhou turned worse dramatically in 2011–2014, leaving far behind the province’ average level. As for economic efficiency, Nanchang, Yingtan, Ji’an and Pingxiang are higher than the province’s average level, whereas Ganzhou, Fuzhou and Yichun are lower than the province’s average level. Nanchang and Pingxiang are excellent in terms of social development, while the remainders are lower than the province’s average for most years of this period, among which the index of Yingtan, Jingdezhen and Fuzhou are constantly below the province’ average line.

In conclusion, all the cities in Jiangxi province have showed positive trends in ecological environment dimension, with the level of majority cities higher than the provincial average level, while social development was the weakness part of ecological civilization in this area. Apart from Nanchang and Pingxiang, all the other cities lied below the province’s average level.

### Evaluation results of the cities

According to the above analysis and dimension weakness number of each city, the 11 cities of Jiangxi province can be roughly divided into the following three types: single-dimension weakness, double-dimension weakness and multi-dimension weakness.

#### 1. Single-dimension weakness

Compared with the average construction level of ecological civilization in Jiangxi province, cities of this type have disadvantages lying in only one single dimension, which indicates that the level of ecological civilization construction is high and the dimension to be improved appears clear. Considering the situation in Jiangxi province, they can be divided into 2 types of disadvantages: resource condition disadvantage and social development disadvantage.

1. Resource condition disadvantage: Nanchang and Pingxiang. Nanchang’s resource condition level is only half of the province’ average, and that of Pingxiang is slightly lower than the province’ average. Compared with other dimensions, the resource condition dimension is greatly limited by the natural resource reserves and the development potential, which shows that the potential of such cities in promotion of ecological civilization construction is limited, and more attention should be paid to the maintenance of advantages in other dimensions.
2. Social development disadvantage: Yingtan and Ji’an. Yingtan is yet to be improved in terms of social development, with the level of that below the province’ average for five consecutive years; In all prefecture-level cities, Ji’an has the best development, and the social development dimension is slightly below the average in the previous three years but it catches up with the average level quickly. Compared with the cities of resource condition disadvantage, this type of cities has unique advantages in terms of resource conditions. Through the efforts of promotion in social development dimension, it is most likely to realize comprehensive ecological civilization as soon as possible.

#### 2. Double-dimension weakness

Cities of this type have disadvantages lying in two main dimensions, indicating that the construction level of ecological civilization in such cities generally needs to be coordinated in both two dimensions, so as to improve the overall level. Considering the situation in Jiangxi province, they can be divided into 2 types: resource condition—social development disadvantage and economic efficiency—social development disadvantage.

1. Resource condition—social development disadvantage: Xinyu and Jingdezhen. The overall ecological civilization construction level of Xinyu reaches the peak among all the cities in 2012. However, it still needs to be improved in terms of resource conditions and social development, for the two indicators are both lower than the province’ average level. The level of Jingdezhen in social development dimension are lower than the province’ average for five consecutive years, and the resource condition dimension is lower than the province’ average after 2012. This type of cities needs to focus on both resource condition and social development dimensions so as to achieve coordinated development, and ultimately to promote the overall level of ecological civilization construction.
2. Economic efficiency—social development disadvantage: Fuzhou and Shangrao. The level of Fuzhou in economic efficiency and social development dimensions are lower than the province’ average, indicating that Fuzhou is in a backward position in the province in terms of economic efficiency and social development. Relying on its advantages in resource conditions dimension, Fuzhou should accelerate the development of economy and society, so as to enhance the comprehensive level of ecological civilization construction. Shangrao has made some improvements in economic efficiency and social development dimension after 2012. This should be continued to maintain a good trend of development as well as eliminate the inferior dimension that still exists.

#### 3. Multi-dimension weakness

Cities of this type have disadvantages lying in three or more dimensions, showing that the level of ecological civilization construction is low in such cities. Efforts should be made in several dimensions at the same time and major attention can be paid to the urgent disadvantages in order to improve the overall level of ecological civilization construction gradually. Considering the situation in Jiangxi province, they can be divided into 2 types: only resource condition advantage and only ecological environment advantage.

1. Only resource condition advantage: Ganzhou. Ganzhou has an advantage in resource condition dimension, but the evaluation results shows that this advantage is gradually decreasing. The level of ecological civilization construction in the city continued to decline after 2012 and the changes occurred mainly in the ecological environment dimension, which indicates that the sustainable decline of ecological environment in Ganzhou has a significant negative impact on the construction level of ecological civilization. Therefore, during the process of taking resource advantages to improve the level of ecological civilization construction, Ganzhou should also attach great importance to the negative effects on the ecological environment brought by resources development.
2. Only ecological environment advantage: Jiujiang and Yichun. In addition to the ecological environment dimension, Jiujiang and Yichun are lower than the province’ average in other dimensions for most years, and the overall level of ecological civilization construction is the lowest of the province. Jiujiang has achieved a certain improvement in economic efficiency dimension after 2013, and the level of Yichun began to grow higher than the province’ average in social development dimension in 2014. This type of cities needs to protect ecological environment quality and take effective measures to improve in all dimensions at the same time, so as to gradually improve the comprehensive level of ecological civilization construction.

## Conclusions and suggestions

### Conclusions

1. **The current overall ecological civilization construction level of Jiangxi province is higher than China’s average (the average level of China is calculated by the combination of AHP and GIS method proposed in this manuscript and all the data source comes from China statistical yearbook.) by 13.23%, but the development of different cities is imbalanced.** The results show that the construction level of ecological civilization in 2014 is 113.23 points, which exceeds the average level of China (100 points) by 13.23%. The average level of all the cities reaches 121.67 points in 2014, with each of them higher than the average level of China. However, the development levels between cities are not balanced, with 9 cities above the province’ average and 2 cities below it (Jiujiang and Jingdezhen), indicating that the two cities have lagged behind a lot than other cities on the level of ecological civilization construction.
2. **The ecological civilization construction level of Jiangxi province at first showed a rise and then a downward trend in 2010–2014.** From 2010 to 2012, it rose from 113.95 points to 117.23 points but showed a downward trend then, it turned out to be 113.23 points in 2014. This indicates that during the study period, the level of ecological civilization construction in Jiangxi province has experienced the change of a first rise and a later decline, and from the perspective of specific cities, different cities show different trends. Among the 7 cities that show upward trends, there are 5 cities that rose continuously (Yingtan, Fuzhou, Ji’an, Pingxiang and Yichun) and 2 cities fluctuated (Jiujiang and Shangrao). Also, among the 4 cities that show downward trends, there are 2 cities that fluctuated (Xinyu and Jingdezhen) and 2 cities fell continuously (Nanchang and Ganzhou).
3. **Different cities of Jiangxi province have various levels in different dimensions of ecological civilization construction, of which the ecological environment dimension is excellent, and the social development dimension is poor.** Among the four dimensions of the evaluation index system, the level of ecological civilization construction in Jiangxi province and its cities underwent the most obvious impact from the ecological environment dimension of corresponding areas. On the whole, the cities of Jiangxi have showed good performance in ecological environment dimension, for during the study period most cities showed to be higher than the average level of the province and only 4 cities has ever appeared to be less than it. Nevertheless, the social development dimension is the weakest in the construction of ecological civilization in Jiangxi. Except for Nanchang and Pingxiang, the remaining 9 cities were lower than the province’s average in most years.

### Policy recommendations

1. **Attach great importance to the imbalance within Jiangxi province and strive to enhance the province’s overall construction level of ecological civilization.** Although the overall construction level of ecological civilization in Jiangxi is higher than the average level of China, it must be clearly recognized that there still exists obvious imbalance in development of the different cities within Jiangxi, and in particular the level of Jingdezhen and Ganzhou is apparently lower than that of other cities. Therefore, we must adhere to the advantages of Jiangxi and give priority to solving the imbalance issue currently existing as well, so as to attain the coordinated development of Jiangxi and to enhance the province’s ecological civilization level as a whole in time.
2. **Pay close attention to the dynamic changes of the construction level of ecological civilization in Jiangxi and reverse the declining trend.** Since 2012, the construction level of ecological civilization in Jiangxi appears to be a declining trend. Therefore, it is of important practical significance for Jiangxi to improve the province’s ecological civilization construction level through scientific and effective ways. In order to achieve this goal, Jiangxi should give full play to the favorable conditions of the province based on the scientific measurement and evaluation of the dynamic changes. Jiangxi should reverse the negative decline trend by starting from the root that influence ecological civilization construction and overall planning, and in time to achieve better development of ecological civilization.
3. **Based on the superior and inferior dimensions of ecological civilization construction, implement the classification guidance of different cities in Jiangxi.** According to the evaluation results of the ecological civilization construction level, each city has different advantages and disadvantages in different dimensions of ecological civilization construction, which should be taken as the important basis for classification guidance. Each type of city should improve its level of ecological civilization construction by highlighting the advantages dimension and making up for the shortages, in order to promote the level of ecological civilization construction accordingly and realize the balanced development goal gradually. Except for Nanchang and Pingxiang, other cities should pay special attention to social development dimension while maintaining the advantages of ecological environment at the same time, so as to achieve the coordinated development of various dimensions.

Although the combination of AHP and GIS can effectively and scientifically evaluate the temporal and spatial changes of ecological civilization, the AHP method based on the preferences, knowledge and judgment of experts and end users, is difficult to uniform the scoring standards of different experts and lacks objectivity. Future research can combine subjective and objective empowerment with GIS to accurately assess the level of ecological civilization construction.
